# Isolation and plasmid characterization of carbapenemase (IMP-4) producing *Salmonella enterica* Typhimurium from cats

**DOI:** 10.1038/srep35527

**Published:** 2016-10-21

**Authors:** Sam Abraham, Mark O’Dea, Darren J. Trott, Rebecca J. Abraham, David Hughes, Stanley Pang, Genevieve McKew, Elaine Y. L. Cheong, John Merlino, Sugiyono Saputra, Richard Malik, Thomas Gottlieb

**Affiliations:** 1 School of Veterinary and Life Sciences, Murdoch University, WA, Australia; 2 Australian Centre for Antimicrobial Resistance Ecology, School of Animal and Veterinary Sciences, The University of Adelaide, SA, Australia; 3 Concord Veterinary Hospital, North Strathfield NSW, Australia; 4 Department of Microbiology & Infectious Diseases, Concord Hospital, NSW and The University of Sydney, NSW, Australia; 5 Centre for Veterinary Education, University of Sydney, NSW, Australia

## Abstract

Carbapenem-resistant Enterobacteriaceae (CRE) are a pressing public health issue due to limited therapeutic options to treat such infections. CREs have been predominantly isolated from humans and environmental samples and they are rarely reported among companion animals. In this study we report on the isolation and plasmid characterization of carbapenemase (IMP-4) producing *Salmonella enterica* Typhimurium from a companion animal. Carbapenemase-producing *S. enterica* Typhimurium carrying *bla*_IMP-4_ was identified from a systemically unwell (index) cat and three additional cats at an animal shelter. All isolates were identical and belonged to ST19. Genome sequencing revealed the acquisition of a multidrug-resistant IncHI2 plasmid (pIMP4-SEM1) that encoded resistance to nine antimicrobial classes including carbapenems and carried the *bla*_IMP-4_-*qacG*-*aacA4*-*catB3* cassette array. The plasmid also encoded resistance to arsenic (MIC-150 mM). Comparative analysis revealed that the plasmid pIMP4-SEM1 showed greatest similarity to two *bla*_IMP-8_ carrying IncHI2 plasmids from *Enterobacter* spp. isolated from humans in China. This is the first report of CRE carrying a *bla*_IMP-4_ gene causing a clinical infection in a companion animal, with presumed nosocomial spread. This study illustrates the broader community risk entailed in escalating CRE transmission within a zoonotic species such as *Salmonella*, and in a cycle that encompasses humans, animals and the environment.

Clinical infections attributed to carbapenem-resistant Enterobacteriaceae (CRE) are a pressing public health issue due to limited therapeutic options. In recent years, studies have reported the emergence and global dissemination of CRE and their public health impact^1^,^2^. The major globally disseminated carbapenemases include KPC, NDM-1, OXA, IMP and VIM^1^,^2^,^3^. Thus far, CRE have been predominantly isolated from humans and environmental samples^1^,^2^,^3^,^4^,^5^. Recent studies have highlighted the global emergence of CREs in both livestock^6^,^7^ and companion animals^8^,^9^,^10^. In particular, recent studies demonstrating the isolation of carbapenemase-producing *Escherichia coli* (NDM-1 and OXA-48) and *Klebsiella pneumoniae* (OXA-48) from clinical infections in dogs^8^,^9^ and have raised concerns about the veterinary use of carbapenems^11^.

In Australia, IMP-4 is the most common carbapenemase detected in Gram-negative bacteria. Thus far in Australia, IMP-4 producing Gram-negative bacteria have been reported predominantly in hospital settings, from both clinical and environmental sources^5^,^12^,^13^,^14^,^15^,^16^,^17^. The *bla*_IMP-4_ gene is considered endemic to Australia and is often carried in a *bla*_IMP-4_-*qacG*-*aacA4*-*catB3* cassette array^13^,^14^,^15^,^16^,^17^. This *bla*_IMP-4_ cassette array is generally found on IncA/C or IncL/M plasmids in New South Wales (NSW) and Victoria^13^,^14^, and IncHI2 or IncL/M plasmids in Queensland^16^,^17^.

A recent study has identified *bla*_IMP-4_ in a range of bacterial species, primarily *E. coli,* from a single, large, off-shore seagull colony in NSW, Australia^18^. This study also identified the *bla*_IMP-4_-*qacG*-*aacA4*-*catB3* cassette array among these isolates, where it was associated with IncHI2, IncA/C, IncL/M or IncI1 replicons. Although numerous serotypes of *Salmonella enterica* were detected in this seagull group, none carried *bla*_IMP-4._ Thus far, there has been no prior report of CREs in either livestock or companion animals in Australia.

In this study we report the first isolation of carbapenemase (IMP-4) producing *S. enterica* from a systemically unwell cat and three additional cats in the same facility. We have also performed for the first time a complete characterization of an IncHI2 plasmid that carries a *bla*_IMP-4_-*qacG*-*aacA4*-*catB3* cassette array and evaluated the heavy metal resistance associated with this plasmid.

## Results

### Bacterial isolation and antimicrobial susceptibility testing

Faecal culturing for *Salmonella* from the index cat resulted in a heavy, pure growth of *S. enterica* serotype Typhimurium (*S.* Typhimurium) on XLD agar. The isolate (SA-2) was resistant to a range of antimicrobials including ampicillin, clavulanic-acid potentiated amoxicillin, clavulanic-acid potentiated ticarcillin, cefazolin, cefoxitin, ceftazidime, ceftriaxone, meropenem, trimethoprim and trimethoprim/sulfamethoxazole and tested positive for the *bla*_IMP-4_ gene. The isolate demonstrated intermediate resistance to tazocin (64 mg/L) and tobramycin (8 mg/L) and was susceptible to ciprofloxacin (S; 1 mg/L), norfloxacin (S; 2 mg/L), cefepime (S; 2 mg/L), amikacin (S; <2 mg/L) and nitrofurantoin (S; 32 mg/L). Of the eight cats tested subsequent to the index case, *S.* Typhimurium was isolated from an additional three cats.

All the isolates (SA-3, 5, 8) had identical resistance profiles to the *S.* Typhimurium isolated from the index cat (SA-2) and were positive for the *bla*_*I*MP-4_ gene. Of these three cats: one had contact with the index case, but remained asymptomatic, another was a kitten that was positive for *Salmonella* spp. before the index case developed diarrhoea. It was located in the same room, but in a separate cage to the index case and had no direct contact. The third case was a young cat which developed diarrhoea, but had no contact with the index case and was not kept in the same room. Following euthanasia of the index case, infection control practices at the cat shelter were reviewed with suggestions to reduce potential cross-transmission. Follow-up monitoring demonstrated absence of *Salmonella* carriage in all animals tested.

### Phylogenetic Analysis

All the isolates (SA-2, 3, 5, 8) belonged to *Salmonella enterica* sequence type 19 (ST19). The phylogenetic analysis of the core genome revealed that the *S.* Typhimurium isolates identified in this study were closely related to internationally reported *S.* Typhimurium ST19 isolates and the *S.* Typhimurium ST19 reference strain (ATCC 14028s) isolated from poultry in the US. Whole genome sequence data revealed that all four isolates belong to sequence type 19 (ST19). At the pan genome level, the four *S.* Typhimurium isolates from this study and the reference ST19 *S*. Typhimurium (ATCC 14028s) strain shared 4879 common genes. The SNP analysis using 4879 common genes demonstrated that all the isolates were identical with the exception of SA-5 which had 2 additional SNPs. All the isolates had 17 SNP differences compared to the reference strain with the exception of SA-5 (19 SNPs).

### Antimicrobial Resistance Genes

Whole genome sequence data revealed the presence of the following antimicrobial resistance genes in the *S.* Typhimurium isolates: β-lactams (*bla*_TEM-1B_, *bla*_OXA-1_), metallo-β-lactams (*bla*_IMP-4_), aminoglycosides (*aac*(*6*′)*Ib-cr, aacA4, strB, strA, aac*(*3*)*-IId*), fluoroquinolones (*aac*(*6*′)*Ib-cr, qnrB2*), macrolides [*mph*(*A*)], trimethoprim (*dfrA19*), sulphonamides (*sul1*), chloramphenicol (*catA2*, *catB3*) and tetracycline (*tetD*).

### Plasmid Characterization

**Figure 1:**
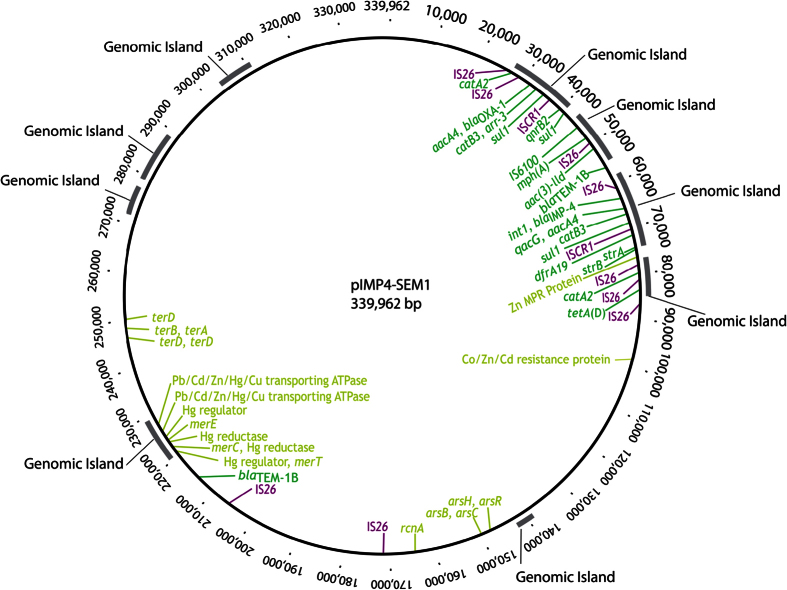
Genomic map of pIMP4-SEM1 carried by carbapenemase-producing *Salmonella enterica* Typhimurium. Significant antimicrobial and heavy-metal resistance associated genes are indicated in green and yellow colours, respectively. Predicted genomic islands are indicated in grey, and insertion sequence positions are indicated in purple.

PacBio sequencing revealed two plasmids; the first a common virulence-associated plasmid, and the second a resistance-associated plasmid. The resistance plasmid (pIMP4-SEM1; GenBank accession number KX810825) was an IncHI2 plasmid (339 kb) that carried all of the antimicrobial resistance genes identified from whole genome sequence analysis (Fig. 1). Conjugation experiments revealed that the carbapenemase-encoding encoding IncHI2 plasmid was easily transferred to *E. coli* J53 with an efficiency of 1.36 × 10^−2^ on MacConkey agar supplemented with ampicillin.

**Figure 2:**
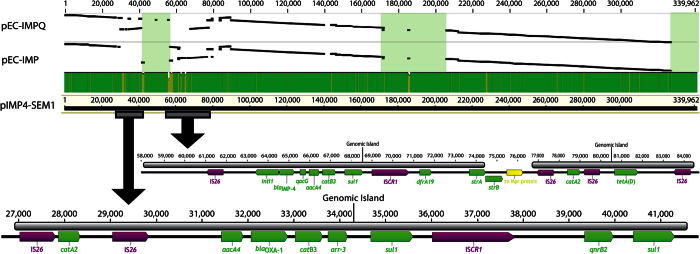
Arrangement of multidrug-resistant regions on pIMP4-SEM1 and genome comparison to pEC-IMP and pEC-IMPQ. Dotplots indicate the percentage identity between pEC-IMPQ and pIMP4-SEM1 and pEC-IMP and pIMP4-SEM1, with increasing plot angles demonstrating homology on the same strand and decreasing plot angles demonstrating homology on opposite strands. Regions showing significant gaps between pEC-IMPQ/pEC-IMP and pIMP4-SEM1 and are indicated by light green boxes. Regions of pIMP4-SEM1 containing the *bla*_IMP-4_-*qacG*-*aacA4*-*catB3* cassette array and the *aacA4- bla*_OXA-1_*-catB3-arr3* cassette array are shown as grey rectangles in the respective positions on the linear genome, with expanded views below.

The resistance plasmid carried a *bla*_IMP-4_-*qacG*-*aacA4*-*catB3* cassette array and an *aacA4-bla*_OXA-1_*-catB3-arr3* cassette array (Figs 1 and 2). In addition to this, the resistance plasmid (pIMP4-SEM1) also contained a number of other antimicrobial resistance genes. Figure 1 shows the arrangement of antimicrobial resistance genes, transposons (IS*26*), integrons (*int1*) and insertion sequence common region (IS*CR1*) among the multidrug resistance region harboured in the cassette arrays on the pIMP4-SEM1 plasmid.

The resistance plasmid was most similar to the IncHI2 plasmids from *Enterobacter* spp. from China, pEC-IMP and pEC-IMPQ (GenBank accession numbers EU855787 and EU855788 respectively) that harboured *bla*_IMP-8_. Blastn analysis of pIMP4-SEM1 against both plasmids revealed a query coverage of 85% and an identity of 99%^19^. Most of the antimicrobial resistance genes were conserved between the three plasmids (Fig. 2). Analysis of the main regions of difference, spanning positions 40,000 to 56,000 of the pIMP4-SEM1 plasmid, revealed a small number of antibiotic resistance genes of low public health significance were present in pIMP4-SEM1 including *mph*(*A*), *aac*(*3*)-*lld* and *bla*_TEM-1B_, but not in pEC-IMP or pEC-IMPQ. The second region, spanning positions 172,000 to 205,000, did not reveal any genes associated with antimicrobial resistance or gene transfer. Both pIMP4-SEM1 and pEC-IMP/pEC-IMPQ plasmids were notable for the presence of a wide range of heavy metal resistance-associated genes. These include genes associated with resistance to arsenic, copper, cobalt, zinc, cadmium, lead and mercury. The majority of these genes were harboured in positions 149,372–166,783 and 221,513–250,546 (Figs 1 and 2). The most significant heavy metal resistance operon was an arsenic resistance operon containing the genes *arsB*, *arsC arsH* and *arsR*. All heavy metal resistance-associated genes were identified in all three plasmids.

The virulence plasmid (pSTV-MU1) (GenBank accession number KX777254) was 93,807 bp in size and carried multiple *Salmonella* virulence factor genes including the *Salmonella* plasmid virulence (*spv*) locus (*spvABCDR*) and the plasmid encoded fimbriae (*pef* ) locus (*pefABCDI*), along with an extensive array of IncF-associated genes. There were no antimicrobial resistance or heavy metal resistance-associated genes present on this plasmid. This plasmid showed 99% identity with query coverages ranging from 97–100% to 26 *Salmonella* virulence plasmids deposited in GenBank.

### Heavy Metal Susceptibility

Heavy metal susceptibility to arsenic, copper, cobalt and zinc was performed to evaluate phenotypic resistance to heavy metals (Table 1). All *S*. Typhimurium isolates and the two transconjugants of *E. coli* J53Az^R^ (SA77, 78) that carried the resistance plasmid were non-susceptible to arsenic with a minimum inhibitory concentration (MIC) of 150 mM. By comparison, the control strains *E. coli* ATCC 25922 and J53Az^R^ were susceptible to arsenic at 3.5 mM and 15 mM, respectively. All the tested isolates were susceptible to cobalt, zinc and copper (Table 1).

## Discussion

In this study we report the first case of clinical infection and carriage of CREs in Australian companion animals and the first detection of *bla*_IMP-4_ positive *Salmonella enterica* serotype Typhimurium from Australia. This study also reports on the genomic characteristics of an IncHI2 plasmid from Australia that carries the *bla*_IMP-4_-*qacG*-*aacA4*-*catB3* cassette array, with demonstrated co-carriage of heavy metal resistance (arsenic).

This is the first report of carbapenem resistance and, more importantly, the presence of a transmissible carbapenemase gene (*bla*_IMP-4_) in a zoonotic species such as *S.* Typhimurium. To date, Australian *S. enterica* strains from livestock remain susceptible to a majority of antimicrobial classes^20^,^21^,^22^ and there have been no reports of CRE among companion animals or livestock in Australia^23^,^24^. However, studies have reported *bla*_IMP-4_ positive CRE among humans, seagulls and in hospital environments, with the predominant bacteria identified as *E. coli, Klebsiella pneumoniae, Enterobacter* spp., and some environmental bacterial genera^5^,^12^,^13^,^14^,^15^,^16^,^17^,^18^. Detection of this extensively drug-resistant *S.* Typhimurium isolate among cats is a significant finding from both an animal health and public health perspective due to the potential for the transfer of these bacteria to other animals and to humans. The acquisition of carbapenem resistance in *S. enterica* is unlikely due to selection pressure from carbapenem use in companion animals since there was no history of such use in the shelter and carbapenems are very rarely used to treat companion animals in Australia^11^. However, co-selection of carbapenem resistance by the use of registered veterinary drug classes (e.g. β-lactams, fluoroquinolones, cephalosporins, tetracylines) may play a role in the acquisition of HI2 like plasmids that carry a wide spectrum of genes encoding resistance to carbapenems and other antimicrobials, including those registered for veterinary use in Australia. With the advent of molecular diagnostics in veterinary pathology, microbiologic diagnoses for enteric infections are increasingly made using PCR based detection methods alone. Without bacterial culture and susceptibility testing of isolates, there is potential for loss of valuable information on emerging critical antimicrobial-resistant pathogens in animals.

Genomic characterization revealed the acquisition of a highly transferable IncHI2 resistance plasmid by a non-host specific, relatively common ST19 clone of *S.* Typhimurium. Acquisition of this plasmid conferred resistance to nine classes of antimicrobials including the critically important classes such as carbapenems (Fig. 1). Furthermore, the isolate demonstrated reduced susceptibility to ciprofloxacin, possibly mediated by two plasmid-mediated quinolone resistance genes (*qnrB2* and *aac*(*6*)*-Ib-cr*), conferring reduced susceptibility to some members of this class and greater propensity to develop mutations in the QRDRs of target chromosomal genes under fluoroquinolone selection pressure.

Recent studies have demonstrated that a highly transferable conjugative IncHI2 plasmid encoding *bla*_IMP-4_ were responsible for increased reports of CRE in Queensland, Australia^16^,^17^. However, limited information is available on the genomic characteristics of IncH12 plasmids that carry *bla*_IMP-4._ The comparative genomic characterisation undertaken in this study revealed that the *S.* Typhimurium IncHI2 plasmid was closely related to IncHI2 plasmids that carry *bla*_IMP-8_ identified from clinical *Enterobacter* spp. isolates in China. Although there are some deletions in the IncHI2 plasmids from China, the significant majority of the antimicrobial resistance and heavy metal resistance-associated genes identified, including the *bla*_IMP-4_-*qacG*-*aacA4*-*catB3* cassette array, are conserved between the three plasmids analysed from Australia (this study) and China (Fig. 2). Similarly, a recent report demonstrated that *bla*_IMP-4_ encoding IncL/M plasmids from Australia shared similar features to several IncL/M plasmids from both Poland and China^14^. While the origin L/M and HI2 plasmids that carry a large spectrum of antimicrobial resistance genes including carbapenem resistance is unclear, the close similarity in genetic content of this plasmid may indicate inter-continental distribution and spread of this critical antimicrobial drug resistance encoding plasmid.

A recent study of CREs isolated from seagulls in NSW Australia has also confirmed the carriage of both L/M and HI2 plasmids containing the *bla*_IMP-4_ cassette array^18^. This raises the question as to whether these plasmids are circulating within the environment in the absence of any antimicrobial selection pressure. One alternative hypothesis, evidenced by the large number of heavy metal resistance-associated genes identified on the IncHI2 backbone, is that exposure of bacteria to heavy metals in the environment may lead to co-selection of plasmids carrying both heavy metal and antimicrobial resistance genes. Although a large number of heavy metal resistance-associated genes were identified on the resistance plasmid (pIMP4-SEM1, Fig. 1), only the arsenic resistance phenotype was observed by MIC testing. This is an interesting finding and demonstrates that the presence of heavy-metal resistance-associated genes alone on a plasmid may not be sufficient for tolerance but may require other genes or host co-factors. The high arsenic MIC (150 mM) identified among the *S.* Typhimurium isolates and transconjugates does, however, demonstrate co-evolution and co-selection of resistance to both critically important antimicrobials and heavy metals. Further studies are required to examine this phenomenon among CRE in a One Health context.

This study illustrates the broader community risk entailed in escalating CRE transmission within a zoonotic species such as *Salmonella*. The findings raise potential concerns for transmission of *bla*_IMP-4_-positive *Salmonella* spp. to humans and the transfer of IncHI2 resistance plasmids to other pathogenic bacterial species such as extra-intestinal pathogenic *E. coli* that share similar ecological niches between humans, companion animals and the environment. There is increasing detection of CRE in Australian hospitals due to carriage of plasmid-encoded *bla*_IMP-4_^5^,^12^. Therefore, it is important to identify the full extent of spread of these plasmids in the environment and to evaluate the potential impact of CRE carriage within companion animals and other animal species including wildlife.

Although, there was initial nosocomial spread between several animals, infection control practices contained the spread of the resistant *S.* Typhimurium within the cat facility. An infectious disease physician (EC) and a veterinary specialist (RM) visited the cattery to critique and modify infection control practices. This One Health approach was successful in controlling the local transmission and spread of potentially zoonotic CRE. The carriage of the *qacG* gene on the IncHI2 plasmid that encodes resistance to quaternary ammonium compounds often used as disinfectants may have infection control implications in veterinary and human clinical settings.

In conclusion, this study for the first time reports on clinical disease due to a *bla*_IMP-4_ positive *S. enterica* in an Australian companion animal and presumed nosocomial spread to other cats in the cat shelter. This study also reveals the full genomic characteristics of a highly transferable IncHI2 plasmid that encodes resistance to carbapenems, range of other classes of antimicrobials and arsenic resistance. Further environmental studies are continuing to examine levels of endemicity of transferable carbapenem resistance in *Salmonella spp.* and other Enterobacteriaceae.

## Methods

### Initial Detection and Screening

Following treatment of an upper respiratory tract infection with doxycycline, a three-year-old castrated male domestic shorthair cat in a cat shelter developed severe haemorrhagic diarrhoea accompanied by malaise and reduced appetite. On admission to a veterinary hospital, a faecal specimen was positive for *Salmonella spp.* by PCR. A rectal swab was therefore sent to the Department of Microbiology Concord Hospital for culture and susceptibility testing. Over the next 48 hours the cat deteriorated rapidly with persistent haemorrhagic diarrhoea and fever, resulting in dehydration and hypovolaemia. A decision was made to euthanase the animal. Rectal swabs were collected from eight cats housed within the same facility, including a cat that had developed mild diarrhoea two weeks after the index case. These cats had been housed in the isolation ward of the shelter when the index cat was symptomatic. Subsequently, rectal swabs were collected from a further nine cats to exclude ongoing *Salmonella* transmission. All methods were carried out in accordance with relevant guidelines and regulations. As samples were for diagnostic purposes, Murdoch University Animal Ethics Committee has issued an Animal Ethics not Required Letter (Protocol number: 270) as per Australian National Health and Medical Research, Animal Research Ethics code.

### *Salmonella* isolation and characterization

Isolation was performed on XLD media (Thermo Fisher Scientific) and typical *Salmonella* colonies identified by MALDI-TOF (Bruker), VITEK^®^ 2 (bioMérieux) biochemical identification and latex agglutination (Thermo Fisher Scientific). The phenotypic antimicrobial resistance profiling was performed on VITEK^®^ 2 (bioMérieux). Once carbapenem resistance was identified a CarbaNP Test was performed (Thermo Fisher Scientific), followed by *bla*_IMP_ gene PCR testing^25^. The *bla*_IMP-4_ characterization was performed using the following in-house PCR-Primers Imp4-F 5′-CACTTGGTTTGTGGAACGTG-3′ Imp4-R 5′-CAATAGTTAACCCCGCCAAA-3′ with a melt peak at 79 to 80.5 °C. Isolates resistant to three or more classes of antimicrobials were classified as multidrug-resistant.

### Conjugation experiments

Transferability of the resistance plasmid was performed by bacterial conjugation using *E. coli* J53Az^R^, as previously reported^26^. The selection of the transconjugants were made on MacConkey’s agar containing sodium azide (100 mg/L) and ampicillin (150 mg/L) or gentamicin (10 mg/L) or imipenem (0.2 mg/L).

### Whole genome sequencing

Whole genome sequencing was performed on all four *S. enterica* isolates (SA-2, 3, 5, 8) using Illumina MiSeq Chemistry. Samples underwent library preparation using Nextera XT DNA library preparation kit according to the manufacturer’s instructions, and sequencing was performed on a MiSeq V3 2 × 300 flowcell. Raw sequence reads were assembled *de novo* using CLC Genomics Workbench v8.5.1. The Nullabor pipeline v1.01 was used to assemble the four Illumina sequenced strains. The reference genome of *Salmonella enterica* serovar Typhimurium ATCC 14028s (accession number CP001363) was used as a reference strain for core genome SNP analysis^27^.

The index case isolate (SA-2) was also subject to PacBio Sequencing. The sequence data was assembled *de novo* using PacBio software and the Quiver programme, and annotation was performed using RAST^28^, with annotation editing performed using Geneious v8.1.2. Pairwise alignment of plasmids was performed using LASTZ^29^.

Screening for antimicrobial resistance genes, MLST and plasmid replicon type was performed using the tools available from Centre for Genomic Epidemiology (http://www.genomicepidemiology.org/). Genomic islands were predicted using IslandViewer 3^30^.

### Screening for heavy metal resistance

Sodium arsenate, copper sulphate, zinc sulphate and cobalt chloride were used to determine the heavy metal susceptibility of *S. enterica* isolates (SA-2, 3, 5, 8), *E. coli* J53Az^R^ and two transconjugants of *E. coli* J53Az^R^ (SA77,78) that carried the resistance plasmid. The metal susceptibility was performed as per Clinical Laboratory Standards Institute Guidelines for performing MIC testing of antimicrobials using Luria-Bertani (LB) broth or agar^31^. Testing was performed in triplicate in both agar dilution and micro-broth dilution. For agar dilution, metals were suspended in varying concentration in LB agar and 10 μL of 5 × 10^5^ CFU/mL of each strain was spotted in triplicate on the plates, followed by incubated for 22 hours at 37 °C. For micro-broth dilution, metals were diluted at varying concentration in LB broth and 10 μL of 5 × 10^5^ CFU/mL added to each well and incubated for 22 hours at 37 °C. *E. coli* ATCC 25922 and J53Az^R^ were used as controls.

## Additional Information

**How to cite this article**: Abraham, S. *et al*. Isolation and plasmid characterization of carbapenemase (IMP-4) producing *Salmonella enterica* Typhimurium from cats. *Sci. Rep.* **6**, 35527; doi: 10.1038/srep35527 (2016).

## Figures and Tables

**Table 1:** Heavy metal susceptibility of *Salmonella enterica* Typhimurium isolates from cats and the two transconjugants of *E. coli* J53Az^R^ that carried the resistance plasmid.

Isolate ID	MIC (mM)			
	Sodium arsenate	Copper sulphate	Cobalt chloride	Zinc sulphate
SA2	150	6	6	3
SA3	150	6	6	3
SA5	150	6	6	3
SA8	150	6	6	3
SA77*	150	3	6	1.5
SA78*	150	6	6	3
*E. coli* J53Az^R^	15	3	6	3
ATCC 25922	3.5	6	3	3

^*^Transconjugants of *E. coli* J53Az^R^ that carried the resistance plasmid.
